# Enhancing Pediatric Tube Weaning with Remote Patient Monitoring: A Pilot Quasi-Experimental Study

**DOI:** 10.3390/nu18060987

**Published:** 2026-03-20

**Authors:** Sarah T. Edwards, Dana M. Bakula, Kristina Nash, Saiyara Baset, Amy Ricketts, Julianne Brogren, Ryan Thompson, Sarah Bullard, Rachel Graham, Janelle Noel-MacDonnell, Brenda Fetter, Lori Erickson

**Affiliations:** 1 Children’s Mercy, Kansas City, MO 64108, USA; dmbakula@cmh.edu (D.M.B.); kmnash@cmh.edu (K.N.); sbaset@cmh.edu (S.B.); jbrogren@cmh.edu (J.B.); rmthompson@cmh.edu (R.T.); rgraham1@cmh.edu (R.G.); jrnoelmacdonnell@cmh.edu (J.N.-M.); bkfetter@cmh.edu (B.F.);; 2 Kansas City School of Medicine, University of Missouri, Kansas City, MO 64108, USA; 3 School of Medicine, University of Kansas, Kansas City, MO 64110, USA; 4 Center for Healthy Lifestyles and Nutrition, Kansas City, MO 64108, USA; sebullard@cmh.edu; 5 Kansas City School of Nursing and Health Sciences, University of Missouri, Kansas City, MO 64108, USA

**Keywords:** feeding, remote monitoring, pediatric, tube weaning, gastrostomy

## Abstract

**Objective:** Feeding-tube weaning is conducted in both inpatient and outpatient settings, with significant logistical, financial, and structural barriers to both approaches. We sought to assess whether remote patient monitoring (RPM), using a mobile application, which would overcome many of these barriers, could be effective in helping patients tube wean. **Methods:** We prospectively enrolled patients with a feeding tube, aged 0–3 years. Enrolled families entered data daily into the remote application. Data were monitored by a nurse and reviewed weekly by a multidisciplinary team. A standard hunger provocation protocol was used, paired with medical, behavioral, oral motor, and nutrition interventions, as needed. We conducted a retrospective chart review to identify a comparison cohort. The chart review was collected first, then compared to the prospective, non-randomized trial of RPM tube weaning. **Results:** The chart review identified 141 children seen with a feeding tube from January 2023–June 2023. Of those, 17 children attempted a tube wean. The post-intervention group consisted of 38 children prospectively enrolled from the same clinic between November 2023–2024. In the pre-intervention group, 41% of the children (7/17) were successful in achieving all calories by mouth and 90% of children (34/38) in the post-intervention group were successful in tube weaning. **Conclusions:** RPM is a feasible and incredibly promising model for feeding-tube weaning in pediatric patients with a wide range of medical comorbidities, including patients with multiple comorbidities. RPM allowed for high-quality medical monitoring and for a dynamic intervention in response to patient data transferred to the medical team in real time.

## 1. Introduction

One in 37 young children have pediatric feeding disorder, which can lead to malnutrition and poor growth [1,2]. A subset of these children, who are unable to meet their nutrition needs through oral intake, require enteral tube feeding. Although feeding tubes are life-saving in many cases, and only placed when essential to patient care, they are burdensome on families, negatively impact child quality of life, and can increase parent stress [3,4].

Feeding tubes are also extremely expensive. US healthcare systems spend approximately USD 183,209 per child for the maintenance of a g-tube over 5 years [5]. Families also report an average of USD 125,645 in lost income associated with caring for their child and an average of USD 523 in medical and supply costs per month not covered by insurance [6]. Half of these parents report on the need to take out loans or reduce spending on basic needs, like food and clothing, to pay for their child’s feeding-related medical care.

The goal for all children who are tube-fed is to discontinue tube feeding as quickly as a child is medically ready to meet their nutritional needs orally. It is well established that the earlier a child is tube weaned, the better [7]. However, there is less consensus on the best approach to tube weaning. Historically, it has been very common to wean children from tube feeding in inpatient or intensive day treatment settings. However, a recent meta-analysis of 40 studies found that both inpatient and outpatient tube weaning programs are effective, as long as they use hunger provocation (i.e., inducing hunger through a gradual reduction in nutrition delivered by feeding tube) and use multidisciplinary teams [8]. Outpatient tube weaning has the significant benefit of being much less expensive and allowing for more equitable access for families who do not have the financial means to take off work or travel to the hospital and stay for days or weeks [9,10]. However, there are significant limitations to outpatient tube weaning. Optimal monitoring involves regular communication between the family and the healthcare team about child weight, hydration, and dietary intake. This can be challenging to accomplish in busy outpatient clinics with high patient volumes. As a result, there has been variable uptake of tube weaning in outpatient settings; outpatient programs tend to have slower rates of weaning [11].

Remote patient monitoring (RPM) may be an ideal tool with which to support the feasibility of outpatient tube weaning. RPM involves the use of technology to collect patient health data from home so that medical teams can review robust amounts of data in real time. The use of RPM in other pediatric populations, such as pediatric cardiology, has resulted in reduced morbidity and mortality [12].

To test the use of RPM for tube weaning, we adapted an existing RPM application for feeding-tube weaning, and conducted a proof-of-concept beta test [13]. Our test patient was successful in transitioning to all calories by mouth within 4 weeks while maintaining age-appropriate growth, with positive family and healthcare team feedback about the use of RPM. Therefore, we sought to prospectively enroll 38 patients with a feeding tube, aged 0–3 years, in a feeding-tube weaning program using RPM. We also identified a comparison cohort via a retrospective chart review. We hypothesized that the use of RPM would improve the feasibility of outpatient tube weaning and demonstrate proof of concept by demonstrating higher rates of tube weaning success compared to outpatient tube weaning without RPM.

## 2. Materials and Methods

The present study was conducted at a large pediatric academic medical center in the Midwest. Patients were all cared for by the hospital’s Interdisciplinary Feeding and Swallowing Program (IFSP), which is a comprehensive feeding treatment program for children with pediatric feeding disorder, serving rural and urban populations across two states. The program is staffed by pediatric gastroenterologists, nurse practitioners, psychologists, speech-language pathologists, occupational therapists, dieticians, nurses, and social workers.

A retrospective chart review of patients weaning with the outpatient standard of care was conducted first for the pre-intervention analysis, then compared to a prospective, non-randomized trial of an RPM tube weaning program (CHAMP version 2.1.9 Children’s Mercy Kansas City, Kansas City, MO, USA). Both studies were approved by the institutional review board with full informed consent provided by parents for participation in the prospective RPM intervention study.

Patients were eligible to attempt a wean from their feeding tube by the IFSP’s clinical standard in both groups if, as follows: (1) the child’s medical conditions were treated/stable; (2) the child’s anthropometrics (i.e., weight, BMIz score) were in the safe range; (3) the readiness of the caregiver (e.g., regular mealtimes, prepared for making changes); and (4) the child had a safe oral intake plan (e.g., if the child was unable to safely swallow thin liquids, they had a safe plan for thickened liquids).

### 2.1. Participants

#### 2.1.1. Pre-Intervention Group

We conducted a retrospective chart review of all patients seen in the IFSP between January 2023–June 2023 who were aged 0–3 years.

#### 2.1.2. Post-Intervention Group

**Figure 1:**
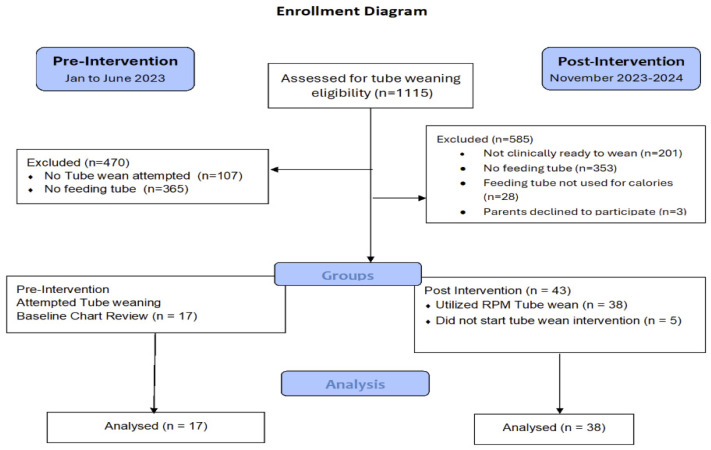
Enrollment diagram.

We screened and recruited patients seen in the IFSP clinic from 11/2023–11/2024, aged 0–3 years, with a feeding tube and goal of weaning to all calories by mouth. Parents of prospectively enrolled, pilot, pediatric participants were approached and enrolled in person or by phone by a trained member of the study team and were taught how to use the RPM platform and a weighing scale. Participant selection at both timepoints is demonstrated in Figure 1.

### 2.2. Measures

For patients in both groups, the measures and methods of tube weaning are defined in Table 1, including the primary outcome. All data were identified via medical chart review; additional data for the post-intervention group were captured by data in the RPM platform. The area deprivation index was calculated using GeoMarker [14,15,16].

### 2.3. Intervention

#### 2.3.1. Pre-Intervention (Retrospective Chart Review of Outpatient Standard of Care) Group

**Figure 2:**
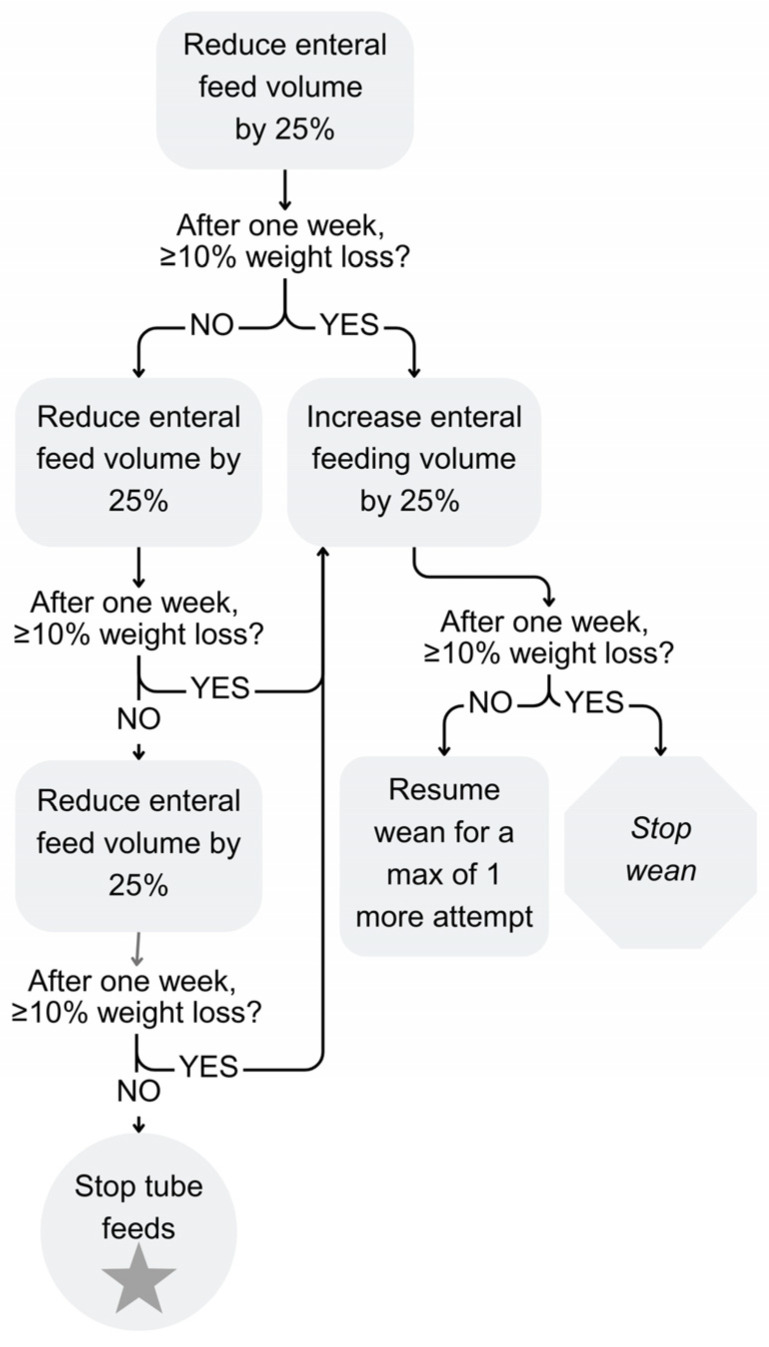
Hunger provocation protocol.

The standard of care in this clinic involves multidisciplinary medical visits every 3 to 4 months with a pediatric gastroenterologist or nurse practitioner, psychologist, dietitian, and occupational or speech therapist. The primary mode of communication between visits is through phone calls and patient portal messages via the electronic medical record. As such, parent-initiated communication relies on the patient portal for safety monitoring and progressing with hunger provocation. The standard-of-care hunger provocation protocol used in this clinic follows established outpatient standard of care over four weeks (Figure 2) [3,8]. This protocol is designed to provoke hunger by reducing the volume of nutrition provided by feeding tube by 25% of the starting volume each week, over four weeks. Each week during the tube wean, child weight is monitored; children only continue to progress with the next volume adjustment as long as they have not lost 10% or more of their starting bodyweight. If a patient does lose 10% or more of their starting bodyweight, feeds are increased and weight is reassessed the following week to determine if the child can resume tube weaning or if tube weaning should be discontinued.

#### 2.3.2. Post-Intervention Group Using CHAMP App^®^

The CHAMP App is an RPM software platform that allows parents to enter daily data about weight, nutrition, hydration, and other health data into an application on their phone, with instant data transfer to the medical team, and the integration of patient data into the electronic medical record. Parents are also presented with a list of concerns in the app that they can select, which will alert the healthcare team, as well as instructions for emergency management. The CHAMP^®^ App was originally developed for use with pediatric patients with congenital heart disease [17], and was adapted by our study team to meet the needs of feeding-tube weaning [13].

During the study, families were asked to enter weekly weights in kilograms and daily reports of intake by mouth, intake by tube, and output through the attainment of all feedings by mouth. The study team reviewed family-entered data weekly and met to discuss patient progress. If there was a lack of entered data, the nurse coordinator reached out to families to collect data and encourage usage of the application, providing suggestions or compromises when necessary. Recommendations from the multidisciplinary team were communicated to the caregiver; together, they collaboratively developed a feeding plan for the week, which was then monitored and tracked through the CHAMP App. The RPM team consists of a physician, nurse practitioners, dietitians, nurses, and a psychologist, as well as a study coordinator, and a research nurse coordinator.

The post-intervention group followed the same hunger provocation protocol as the outpatient standard-of-care group. Once a patient was successfully weaned, they were monitored for four additional weeks before being discharged from RPM.

### 2.4. Analysis Plan

We conducted a priori sample size calculation to determine the sample size needed to detect a meaningful difference across groups. We used established success rates of 47% in outpatient tube weaning [18] and a predicted RPM success rate of 80% to determine that we would need a sample size of 38 patients in the post-intervention group, accounting for 20% attrition (Sample Size Calculator, 2023), to be powered at 0.8 with α = 0.05.

Descriptive statistics were used to describe demographic and medical variables for each group. Groups were compared using SPSS v. 29 and SAS Version 9.4. Due to the sample size, comparisons were made with Fisher’s exact tests and Mann–Whitney U tests, respectively. Additional secondary analyses were completed for the post-intervention group, including frequency of tube progression and a Kaplan–Meier survival estimate to assess days to success with all feedings by mouth. Mobile application engagement and utilization metrics were operationalized through measures of RPM data feeding entry and weekly weights [19]. To mitigate potential innovator bias, statistical analyses were intentionally conducted by the team biostatistician who did not have a conflict of interest.

## 3. Results

The demographic and medical characteristics of the pre-intervention group (outpatient standard of care) and the post-intervention (RPM) group are delineated in Table 2. The retrospective chart review identified 141 children seen in the IFSP with a feeding tube during the 6-month period. Of that group, 17 children attempted a tube wean. The post-intervention group consisted of 38 children. Enrollment based on clinical clearance to attempt a wean from a feeding tube did not differ significantly across time periods (13.7%, 17/124 v. 15.4%, and 38/247, respectively).

There were no differences in demographics or clinical complexities across the two groups. There were differences between the two groups with respect to the subspecialists they were followed by. Over a third of the children in both groups were followed by cardiology; however, there was a higher proportion of children with complex cardiac disease in the post-intervention group (26% vs. 0%, *p* = 0.002).

We found that it was common for children in both groups to be prescribed a medication for feeding- or gastroenterology-related concerns and did not see differences in the rate of medication use across the groups. In the full cohort, approximately one third (32%) were prescribed cyproheptadine, 6% erythromycin, 10% esomeprazole and ferrous sulfate, 13% docusate, 25% MiraLAX, 17% omeprazole, and 15% lactulose.

### 3.1. Primary Outcomes

In the pre-intervention group, 41% of the children (7/17) who attempted a wean were successful in achieving all calories by mouth and 90% of children (34/38) in the post-intervention group were successful. When comparing the probability of successful tube wean across children in both groups based on RPM, demographics, and medical variables (Table 3), exposure to the post-intervention group and a swallow study where honey-thickened liquids were recommended were of greater significance for successful weaning.

### 3.2. Post-Intervention Group Using the CHAMP App

#### 3.2.1. Time to Wean

**Figure 3:**
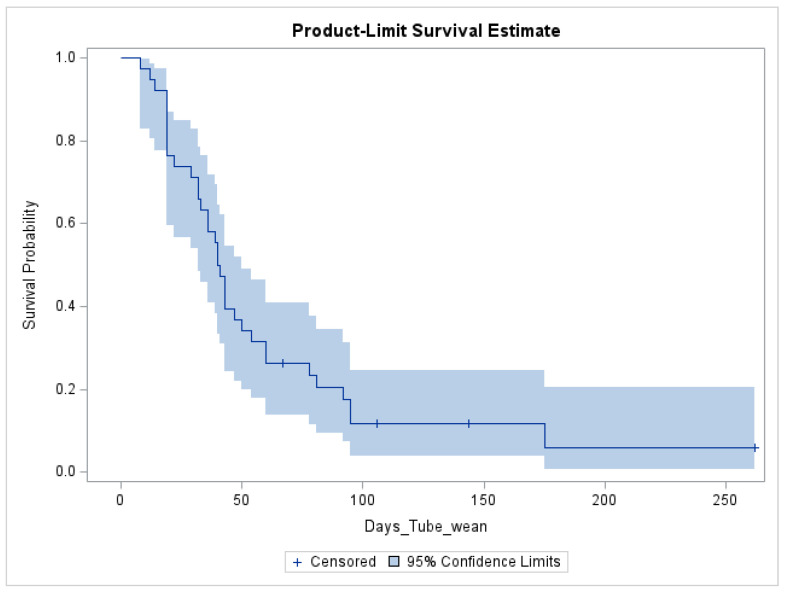
Product-limit survival curve.

The median time to successful wean was 40 days ([19, 54] days) compared to those not successful, at 125 days ([87, 203], *p* = 0.007). Since only four children did not wean in the post-intervention group, we were unable to assess factors predictive of treatment failure in this group due to limited power. However, descriptively, we found that the reasons for treatment failure in the four children who did not wean in the post-intervention group were affected by parent concerns about weight loss (n = 1), although they did not reach the 10% threshold, and external factors including social challenges (n = 2) and competing medical priorities (i.e., surgery; n = 1). See Figure 3 for a calculated statistical survival estimate for time to a successful tube wean. This approximation indicates that after 54 days, if the child has not reached all calories by mouth, their likelihood of success in the tube wean is low.

#### 3.2.2. Tube Weaning Progression

The median number of cuts to the volume of nutrition delivered by tube between the starting volume and the ending volume was four (95% CI [3, 5]). Most patients were able to only make cuts without needing to add back in any volume. However, four patients had their feeding-tube volume increased during the monitoring period due to weight loss or inadequate feeding by mouth, for a total of eight increases. Two of these patients eventually were successful in weaning during the study. All but one of the increases were parent-driven and not initiated by the medical team.

#### 3.2.3. Weight Trajectory

No patients had more than 10% weight loss from baseline. Three of four patients who needed increased feeding were unable to get back to all calories by mouth. Many patients were able to either sustain their starting weight or gain weight during tube weaning. See Supplementary Figure S1 for weight curve graphs.

#### 3.2.4. Engagement

All families entered at least one RPM data point during the study period. Over an average of 12 (SD = 7) weeks, the mean adherence to weekly weight data entry was 96% across the 38 patients who attempted a tube wean with the RPM system.

#### 3.2.5. Red Alerts and Clinical Concerns

There was a total of 66 red flags by 22 families, with 42% of children having no reports of red flags or concerns during the RPM monitoring. The app features a list of “red flags” or possible clinical concerns that a family may encounter during tube weaning and would warrant contact with the medical team. There were five non feeding-related hospitalizations/procedures (three unplanned and two planned) during the tube wean period, where feeding changes were paused. See Table 4 for a list of all red flags reported during the study period, their route of communication, and primary recommendations.

#### 3.2.6. Adverse Events

There were no adverse events during the study period that were deemed to be related to the study treatment. Adverse events monitored included weight loss greater than 10% of starting weight and hospitalizations related to feeding.

## 4. Discussion

Feeding tubes are lifesaving; however, they are also extremely expensive to both the healthcare system and families. Prolonged feeding-tube use negatively impacts child quality of life and delays oral motor development. We sought to assess the feasibility of using RPM for tube weaning and test the proof of concept that RPM could improve the quality of outpatient tube weaning by facilitating regular and efficient communication between the patient and provider. We found that RPM was highly feasible to implement with this population. Regarding proof of concept, we found that the implementation of RPM in an outpatient tube weaning program was associated with improved success rates—ranging from 41% to 90%—in achieving all calories by mouth. Patients who used RPM were able to successfully wean in an average of 40 days, with only four patients unsuccessful in achieving all calories by mouth by the end of the study, and without any patients experiencing adverse effects, including weight loss beyond 10% of their starting bodyweight. This demonstrates strong preliminary evidence that RPM for tube weaning warrants additional exploration through prospective, randomized clinical trials. Overall, this exploratory pilot of RPM demonstrated strong potential for successful, timely tube weaning, and was able to overcome barriers to both inpatient and outpatient tube weaning programs [8].

Patients who used the RPM platform were able to wean in a timely manner, relative to our retrospective outpatient standard-of-care cohort, as well as others [11,18]. However, if patients were unable to wean within the first 55 days, they were unlikely to be successful. Although inpatient programs are typically brief and time-limited [8], outpatient tube weaning may last for months in some programs [11]. Clinicians may find it valuable to re-assess goals if tube weaning in an outpatient program persists for longer than 55 days. We were unable to identify other within-person predictors of unsuccessful tube weaning in the post-intervention group due to the low rate of treatment failure; thus, no adjusted statistical models were conducted. However, it was notable that half of the reasons for tube weaning failure were due to social barriers. Therefore, it is important to discuss family goals for tube weaning prior to initiating, and throughout, the process.

Patients in both treatment groups had complex medical needs and followed an average of six pediatric subspecialists. However, medical complexity did not impact the likelihood of success. It is common for medical providers and families to feel wary of tube weaning children who have multiple medical comorbidities. However, the earlier that a child tube weans, the more likely they are to be successful; therefore, providers should not discount a patient from weaning due to complexity [7].

Our RPM model provided parents with the opportunity to enter data about a child’s oral intake daily, enter weights weekly, upload videos of the child eating, and report red flags 24/7 with a daily data review from a trained nurse coordinator and weekly data review via virtual rounding by the multidisciplinary team. This supported dynamic opportunities for the early identification of concerns and associated interventions. This level of close contact has been shown to help families and medical providers feel more confident with medical management from the homes of complex patients [17]. The same confidence may also enable future clinicians to feel more comfortable when referring more and more complex patients for earlier tube weaning intervention.

Prior studies have found that RPM is effective regardless of socioeconomic status, which was replicated in this study [12]. We also found that RPM improved access to care for vulnerable populations. Pre-intervention, we did not wean any children whose families did not speak English. Using RPM, we successfully weaned three children whose families spoke Spanish, as the app is available in several languages. The combined benefit of the reduced cost burden and reduced barriers to care make RPM a particularly compelling option for the future of tube weaning.

There are several limitations to this study. As of 2026, the CHAMP App has been used in more than 1400 cardiac patients across nine hospitals and in 29 states since 2015. While this focused pilot study was the first published for tube weaning, weight management in high-risk, complex infants is not new for this care model. Consistent with prior meta-epidemiological findings, early single-center studies tend to report larger intervention effects relative to subsequent multi-center trials, likely reflecting small-study effects, publication bias, and methodological variability. This should be considered when reviewing findings of this publication. This was an exploratory, non-randomized pilot trial; a future randomized controlled trial with a robust sample of patients in both the active treatment group and control group, with a longer follow-up period is essential.

Though we found higher success rates in the RPM group, the research design does limit the ability to inform causal inference. The focus on tube weaning may have also impacted a Hawthorne effect, with an increased focus on hunger provocation across two time periods. All patients seen in the feeding clinic across both time periods were screened and reviewed for tube weaning to reduce any prospective selection bias; however, there were no explicit controls for selection bias in the retrospective cohort, as these patients underwent tube weaning prior to the implementation of this study. We do acknowledge that the healthcare teams’ comfort with the tube weaning process via RPM may have increased the indication bias to attempt to medically clear more children for tube weaning. This, however, would have potentially decreased the chance of success of all calories by mouth in the RPM group versus increasing the chances of improved outcomes. This study was conducted at a single site in the Midwest of the Unites States, with mostly English-speaking families, which may limit the generalizability of findings. Future studies should be conducted at multiple sites to assess the differential impact of the intervention based on location-specific factors. Additionally, the RPM group did have a slightly lower % EER met by tube at baseline relative to the retrospective cohort, which may have impacted findings. RPM has the potential to increase scalability; however, the cost, including up-front costs and long-term sustainability costs, should be further explored when additional efficacy data are established. Additionally, this type of research relies on access to RPM technology and a nurse coordinator to review data daily and communicate with families about it, which may not be feasible in all settings. Lastly, it should be noted that, without adjusted statistical analysis completed, there may be factors (i.e., clinical severity, caregiver characteristics, nutritional status, and, as mentioned above, social barriers) that influence the failure to wean. These factors may have differed across groups, which is not possible to control in a non-randomized design such as this one. It is possible that differences in treatment outcomes between the RPM group and retrospective cohort are due in part due to inherent differences between the two cohorts, or related temporal or contextual factors, making the need for a prospective randomized trial all the more pertinent. Greater insight into these factors will be possible in a future larger-scale study with a larger sample size and adjusted statistical analysis controlling for patient and/or caregiver factors. Regardless of these limitations, this study revealed that RPM has great promise for feeding-tube weaning, as these results are a strong signal of RPM feasibility and proof of concept for tube weaning.

The next step, in line with the ORBIT model for clinical trial development [20], will be a prospective, randomized clinical trial of RPM compared to the outpatient standard of care, with long-term follow-up. Although adequately powered based on previous studies in the literature, the small sample size of our cohort limited the statistical methodology beyond univariate and bivariate analysis. Larger study samples may better explain the variance and nuances of the complex care surrounding tube weaning from a feeding clinic. Future directions should also include longer-term follow-up post tube weaning to assess a patient’s abilities to be successful with tube weaning after the acute post-wean phase, as it will be critical to assess the success of this intervention in leading to sustained nutritional and developmental progress.

## 5. Conclusions

In sum, we found that RPM could be a feasible model for feeding-tube weaning in pediatric patients, even in patients with several medical comorbidities. RPM allowed for high-quality medical monitoring and dynamic intervention in response to patient data that were transferred to the medical team in real time, and safe patient outcomes without excessive weight loss. Future studies should evaluate the efficacy of this approach through large, randomized clinical trials to rigorously assess efficacy.

## Figures and Tables

**Table 1:** Measures and methods across groups.

	Pre-Intervention Group	Post-Intervention Group
Baseline Measures		
Demographics and medical history	Demographic and medical data were obtained from the medical record, including age, gender, medical history, number of hospitalizations, medications, estimated energy requirements (EER), and insurance type.	
Area deprivation index (ADI)	Patient zip code is used to calculate the ADI using local data on fractions of assisted income, high school education, no health insurance, poverty, vacant housing and the area median income. The ADI scale is 0–1, with 0 indicating less deprivation and 1 indicating the highest level of deprivation. This measure is used as a proxy of socioeconomic status.	
Anthropometrics	Baseline and end-of-wean anthropometric data were obtained from the medical record for patients in both groups, including weight (kilograms), length (centimeters), head circumference at birth and tube weaning start, weight for age, weight z-scores, weight percentiles, body mass index (BMI), and BMIz.	
Outcome Measures		
Tube weaning primary outcome: % EER	As the primary outcome metric, rate of success in a dichotomous (yes/no) to 100% of calories by mouth taken for age-appropriate growth, estimated by the child’s EER. EER is calculated by the feeding team nutritionist in clinic or the study team nutritionist.	
Weight monitoring	Retrospective review of parent-reported weights through phone calls, clinic visits, and patient portal messages via the electronic medical record.	RPM mobile data entry, weekly weights, and clinic visits.
Tube weaning duration (days)	Tube weaning initiation, time to complete wean in days	
Weaning-related data	Data collected via retrospective review of (1) parent-reported pertinent updates via phone calls, clinic visits, and patient portal messages, and (2) medical team communications logged in the medical record about next steps for tube weaning progression.	Data collected via RPM platform and logged prospectively in a research log if reported via phone, office visit, or patient portal message. Data obtained included feeding volumes by mouth and tube and videos of eating.
Tube weaning concerns	Parent-reported concerns during tube weaning were obtained via retrospective chart review of phone calls, office visits, and patient portal messages.	Red flags were prospectively monitored in the RPM app. The app provided parents with a list of common concerns during tube weaning. Parents could select a concern, which would immediately alert the healthcare team. Red flags reported via other routes (e.g., phone call, portal message) were also prospectively tracked.
Parental non-adherence to tube weaning	No proactive non-adherence management	If there is a lack of entered data, the nurse coordinator reaches out to families to collect data and encourage usage of the application, providing suggestions or compromises when necessary.
Intervention Methods		
Tube weaning protocol	Hunger Provocation Protocol (Figure 2)	
Healthcare team	Patients attended multidisciplinary medical visits every 3 to 4 months with a pediatric gastroenterologist or nurse practitioner, psychologist, dietitian, and occupational or speech therapist.	Patient data were reviewed M-F by a research nurse coordinator. A multidisciplinary team reviewed all patient data together via virtual rounds. The team included a pediatric GI physician, psychologist, speech therapist, nurse practitioners, dietitians, and nurses, as well as a study coordinator and a research nurse coordinator.
Route of communication	Parent-initiated communication to the feeding team.	RPM initiated data by parents with a weekly proactive nurse coordinator message for each family, weekly healthcare team meetings.
Concern reporting	Common concerns were reviewed by the medical team with parents prior to starting tube weaning, then parents contacted the feeding team if they noticed concerns.	The RPM app provides parents with a list of common concerns during tube weaning. Parents could select a concern, and the healthcare team was alerted. Red flags could also be reported via other routes (e.g., phone call).

Note. Abbreviations: EER, estimated energy requirements.

**Table 2:** Descriptive patient characteristics of the children who attempted a tube wean.

	Pre-Intervention Group (n = 17)	Post-Intervention Group (n = 38)	*p* Value
Male sex assigned at birth, % (n)	53% (9)	42% (16)	0.46
Race, % (n)			0.38
White	77% (13)	82 (31)	
Black/African American	6% (1)	8% (3)	
Multi-racial	6% (1)	5% (2)	
Asian	6% (1)	0%	
Native Hawaiian/Pacific Islander	6% (1)	0%	
Hispanic	0%	5% (2)	
Hispanic Ethnicity, % (n)	6% (1)	16% (6)	0.42
Primary language English, % (n)	100% (17)	92.1% (35)	0.54
Mother is primary feeder, % (n)	100% (17)	92.1% (35)	1.00
Distance from hospital to home in miles, median (IQR)	24.95 (110.03)	46.55 (119.19)	0.49
ADI, median (IQR)	0.318 (0.135)	0.316 (0.204)	0.95
Median yearly income by address in USD, median (IQR)	55,899 (18,145)	55,998 (35,213)	0.90
Gestational age in weeks, median (IQR)	38.6 (6.0)	38.0 (4.0)	0.92
Birth weight in kg, median (IQR)	2.8 (1.5)	2.9 (1.1)	0.79
NICU stay, % (n)	71% (12)	63% (24)	0.59
Number of pediatric subspecialists, median (IQR)	6.0 (6.0)	6.0 (4.0)	1.00
Number of hospitalizations before tube weaning, median (IQR)	3.0 (1.0)	3.0 (3.0)	0.73

Note. Abbreviations: ADI, area deprivation index; IQR, interquartile range; NICU, neonatal intensive care unit.

**Table 3:** Comparisons based on tube weaning success across both treatment groups.

	Not-Weaned (n = 14)	Weaned (n = 41)	*p* Value
RPM using CHAMP App % (n)	29% (4)	83% (34)	<0.001
Blended diet % (n)	29% (4)	32% (13)	1.00
Swallow study result: honey-thickened liquids	29% (4)	2% (1)	<0.001
Age at initiation of weaning attempt (months), median (IQR)	27.18 (12.1)	26.57 (19.6)	0.99
Weight for length z-score at tube wean, median (IQR)	−0.2 (−0.3)	0.7 (−0.8)	0.49
Length of enteral tube duration before wean attempt (months), median (IQR)	24.0 (13.1)	21.0 (24.0)	0.76
Gastrostomy tube at time of weaning % (n)	86% (12)	81% (33)	1.00
Starting EER % supported by enteral tube feedings, median (IQR)	90.4 (37.7)	58.2 (65.3)	0.08
Duration to tube wean (days) median (IQR)	161 (162)	43 (52)	<0.001
Number of feeding changes by a healthcare team member, median (IQR)	2.0 (1.0)	4.0 (2.5)	0.07

Note. Abbreviations: EER, estimated energy requirements; IQR Interquartile Range.

**Table 4:** Red flags for tube weaning by remote patient monitoring model of care with recommendations.

Type	Frequency	Route of Communication		Primary Recommendation	
Eating less than they used to	35% (23)	RPM	57% (13)	Review and reassure	62% (8)
				GI clinic	7% (1)
				Medication adjustment	7% (1)
				GI Psychologist	7% (1)
				Increased mobile app data entry	15% (2)
		Parent called	26% (6)	Review and reassure	33% (2)
				Outpatient feeding change	33% (2)
				PCP	17% (1)
				Outpatient lab	17% (1)
		Team noted	17% (4)	Review and reassure	25% (1)
				Outpatient lab	25% (1)
				Medication adjustment	25% (1)
				Outpatient 48 h calorie count	25% (1)
Child having non feeding-related Illness	24% (16)	RPM	6% (1)	Review and reassure	100% (1)
		Parent called	75% (12)	Review and reassure	67% (8)
				Emergency room	17% (2)
				PCP	8% (1)
				Increased mobile app data entry	8% (1)
		Team noted	19% (3)	Review and reassure	67% (2)
				PCP	33% (1)
Losing weight	9% (6)	Team noted	100% (6)	Outpatient 48 h calorie count	50% (3)
				Medication adjustment	17% (1)
				Outpatient feeding change	17% (1)
				Increased mobile app data entry	17% (1)
Ability to feed in the last 24 h was …	7% (5)	RPM	100% (5)	Review and reassure	80% (4)
				Medication adjustment	20% (1)
Family non-adherence to feeding plan	4% (3)	Team noted	100% (3)	Outpatient feeding change	33% (1)
				GI Clinic evaluation	33% (1)
				Increased mobile app data entry	33% (1)
Vomiting after every feed	3% (2)	Outside Provider *	50% (1)	GI Clinic evaluation	100% (1)
		RPM	50% (1)	Review and reassurance	100% (1)
No stool for 3 days	3% (2)	Parent called	100% (2)	Medication adjustment	100% (2)
Refusing most or all bottles	3% (2)	RPM	50% (1)	Increased mobile app data entry	100% (1)
		Team noted	50% (1)	Review and reassurance	100% (1)
Concerns with feeding tube	3% (2)	Parent called	100% (2)	Emergency room	50% (1)
				Review and reassurance	50% (1)
Appearing uncomfortable when feeding	3% (2)	RPM	50% (1)	Review and reassurance	100% (1)
		Parent called	50% (1)	GI psychologist	100% (1)
Symptoms worsening with the prescribed plan	2% (1)	RPM	100% (1)	Review and reassurance	100% (1)
Choking gagging or coughing	2% (1)	RPM	100% (1)	Review and reassurance	100% (1)
Taking longer to finish meals	2% (1)	RPM	100% (1)	Review and reassurance	100% (1)
Total Red Flags	66				

Note. RPM = concern reported via RPM App; Parent Call = Parent called concern to report via phone or voicemail; Team noted = Medical team identified the concern based on data review. * Follow-on call.

## Data Availability

Deidentified individual participant data (including data dictionaries) will be made available, in addition to study protocols, the statistical analysis plan, and the informed consent form. The data will be made available upon publication to researchers who provide a methodologically sound proposal for use in achieving the goals of the approved proposal. Proposals should be submitted to sedwards1@cmh.edu.

## Supplementary Materials

The following supporting information can be downloaded at: https://www.mdpi.com/article/10.3390/nu18060987/s1, Figure S1: Weight trajectory of each RPM tube wean subject. SF1. Weaned Status; SF2. Weaned Status, SF3. Weaned Status, SF4. Weaned Status, SF5. Weaned Status, SF6. Weaned Status, SF7. Weaned Status, SF8. Weaned Status, SF9. Weaned Status, SF10. Weaned Status, SF11. Weaned Status, SF12. Weaned Status, SF13. Weaned Status, SF14. Weaned Status, SF15. Weaned Status, SF16. Weaned Status, SF17. Weaned Status, SF18. Weaned Status, SF19. Weaned Status, SF20. Weaned Status, SF21. Weaned Status, SF22. Weaned Status, SF23. Weaned Status, SF24. Weaned Status, SF25. Weaned Status, SF26. Weaned Status, SF27. Weaned Status, SF28. Weaned Status, SF29. Weaned Status, SF30. Weaned Status, SF31. Weaned Status, SF32. Weaned Status, SF33. Weaned Status, SF34. Weaned Status, SF35. Weaned Status, SF36. Weaned Status, SF37.

## Abbreviations

The following abbreviations are used in this manuscript:

ADI	Area Deprivation Index
BMI	Body Mass Index
EER	Estimated Energy Requirements
IFSP	Interdisciplinary Feeding and Swallowing Program
ORBIT	Obesity-Related Behavioral Intervention Trials
RPM	Remote Patient Monitoring
