# Supplementary figures and images for: Enhancing Pediatric Tube Weaning with Remote Patient Monitoring: A Pilot Quasi-Experimental Study
Source: Nutrients. 2026 Mar 20;18(6):987. doi: 10.3390/nu18060987 (PMC13029489; doi:10.3390/nu18060987)

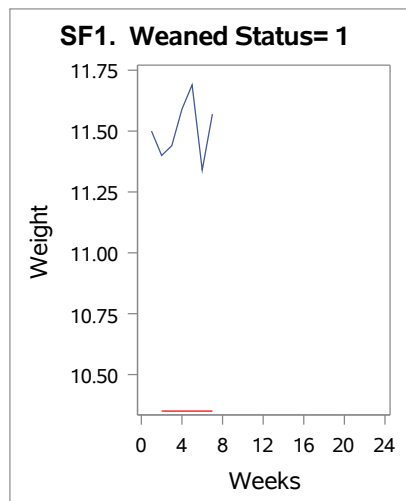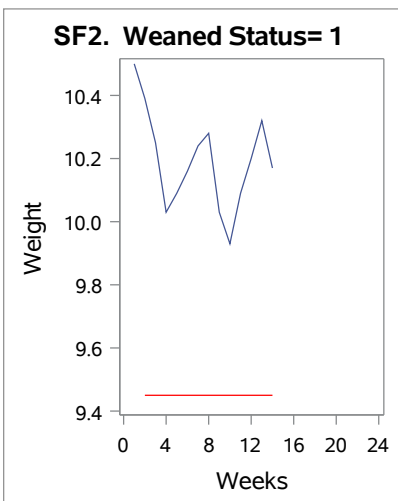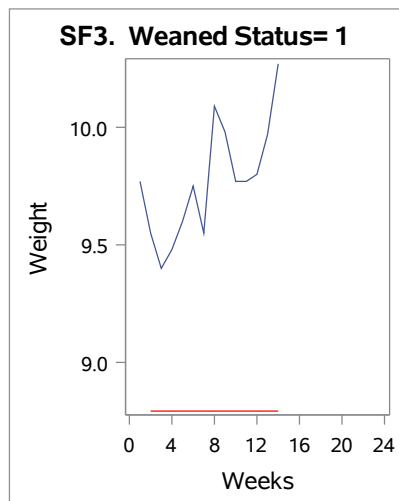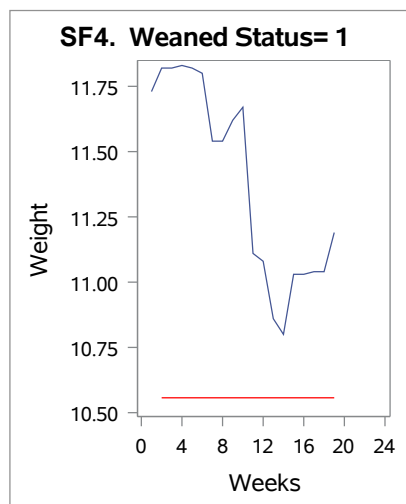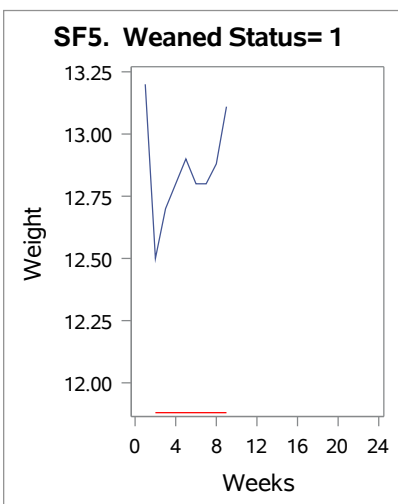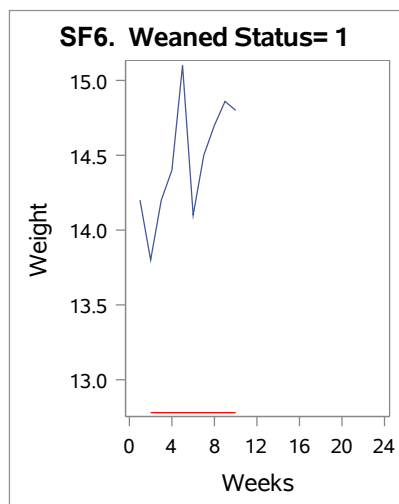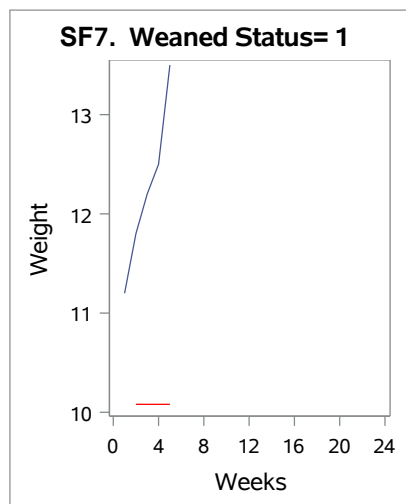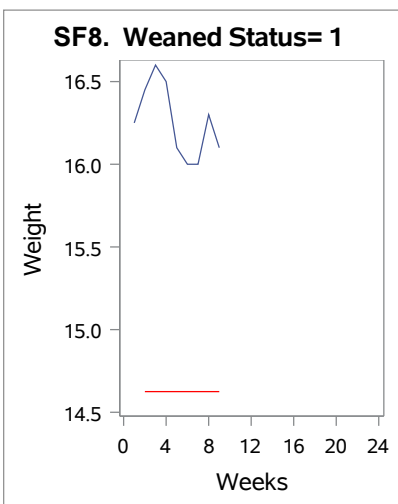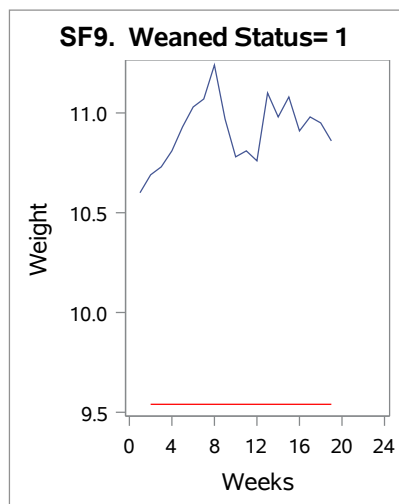

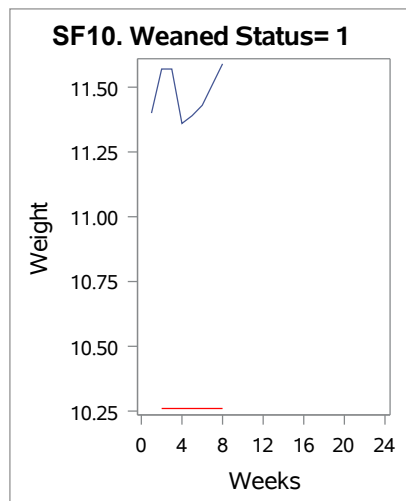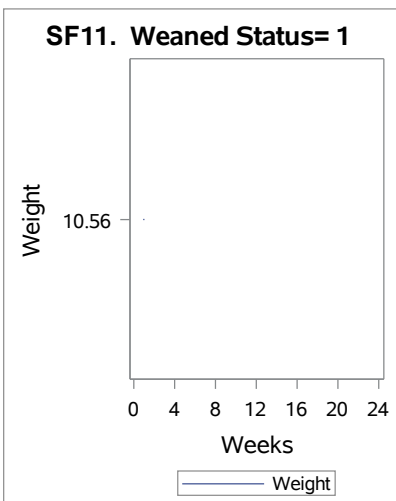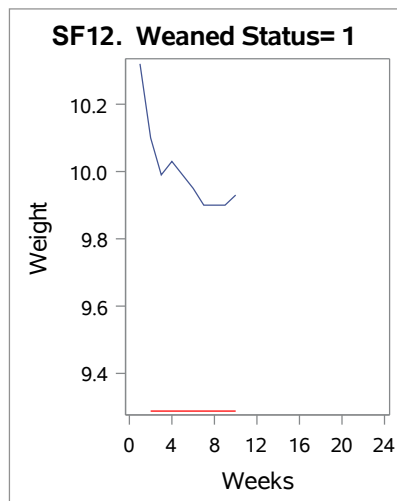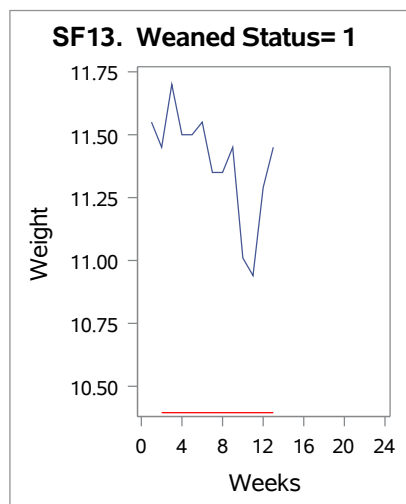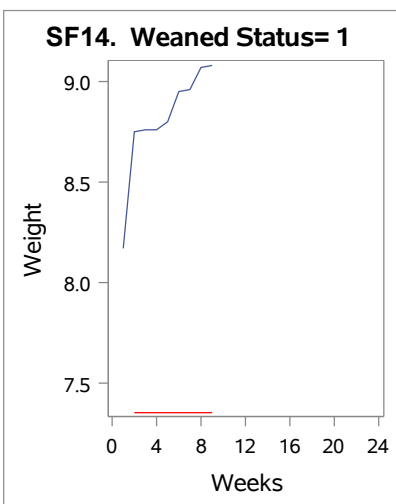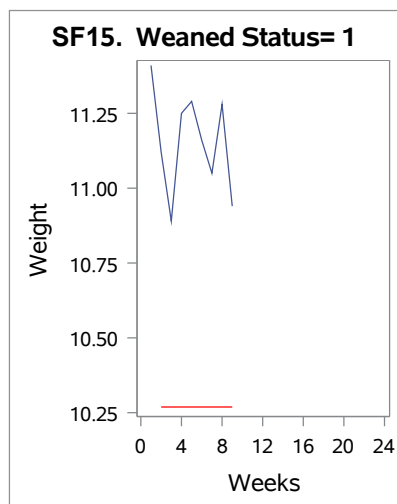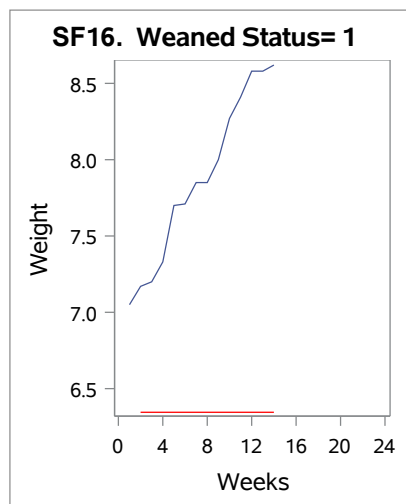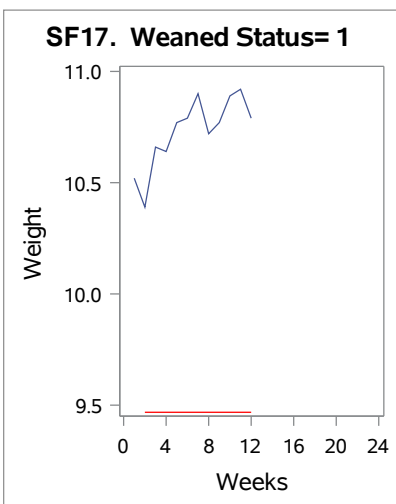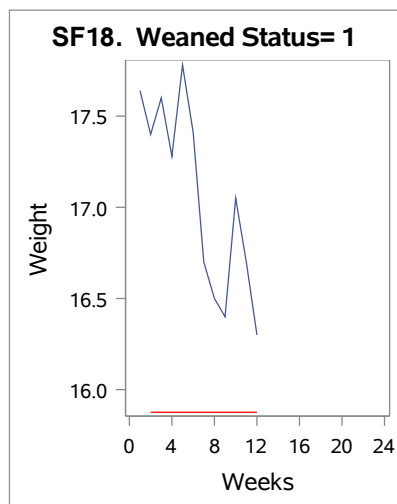

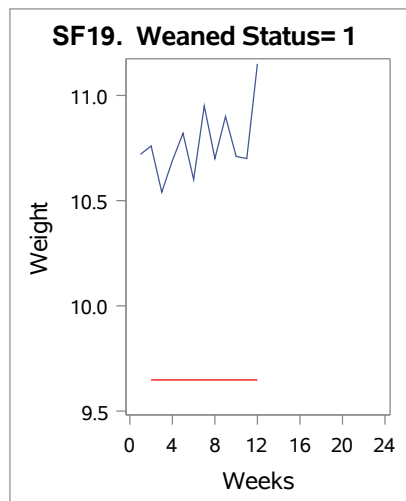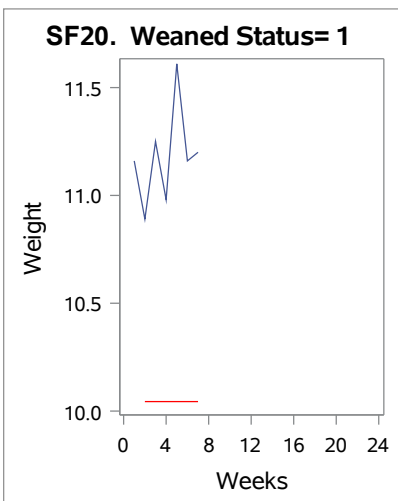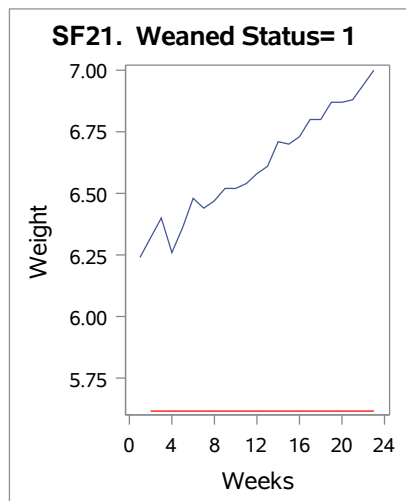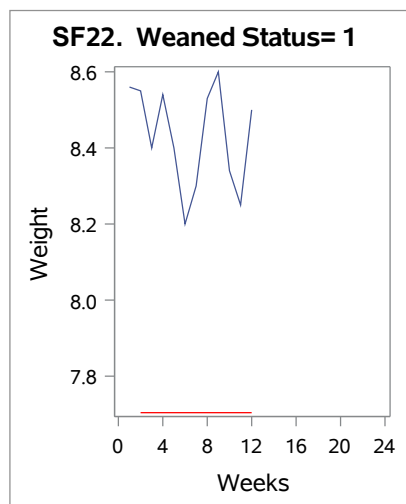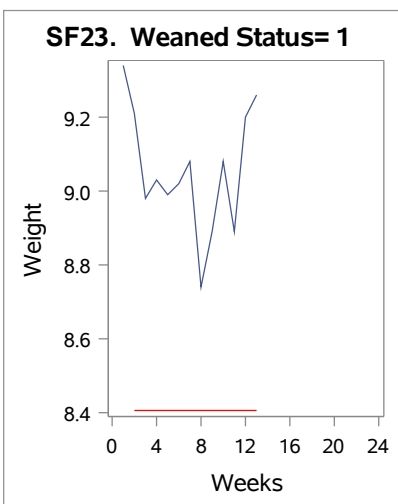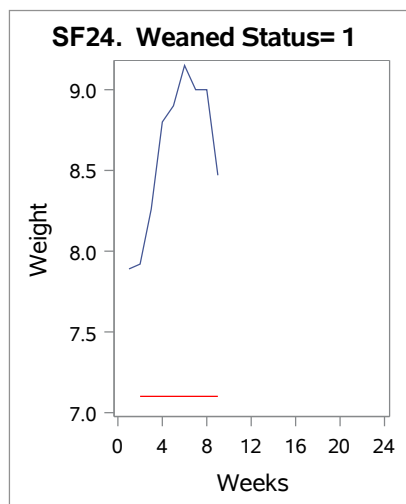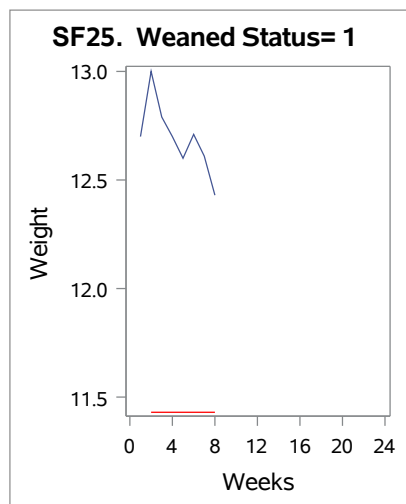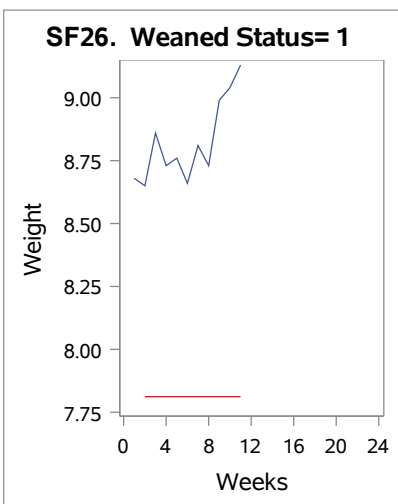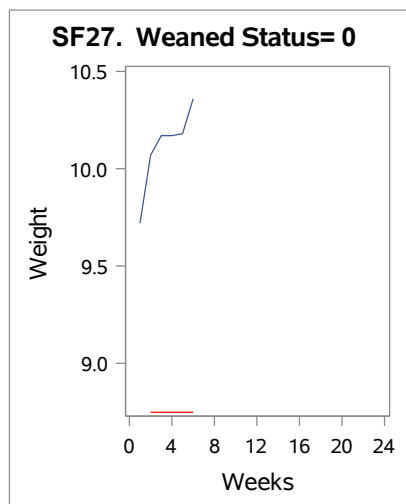

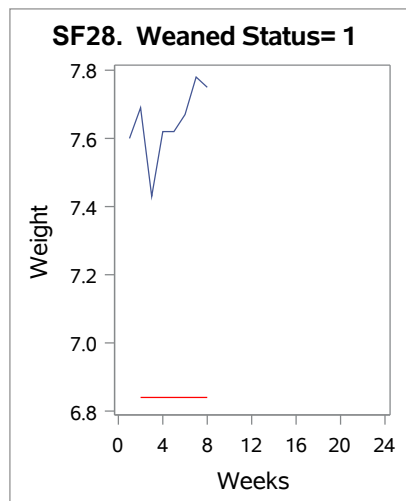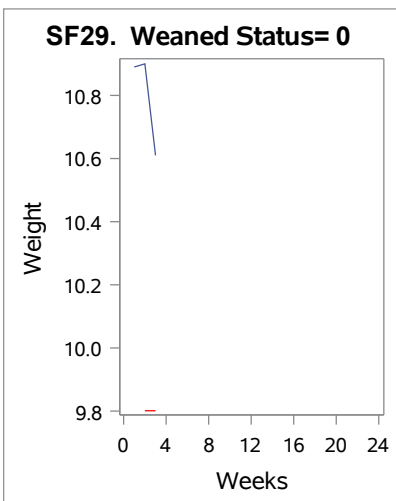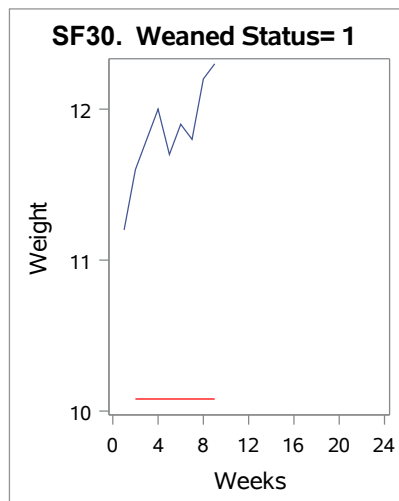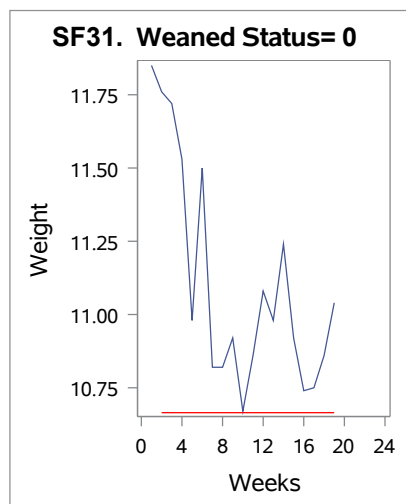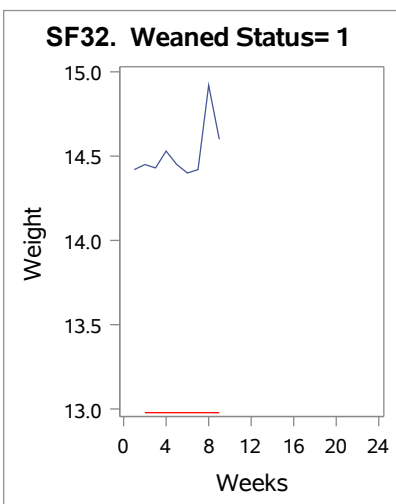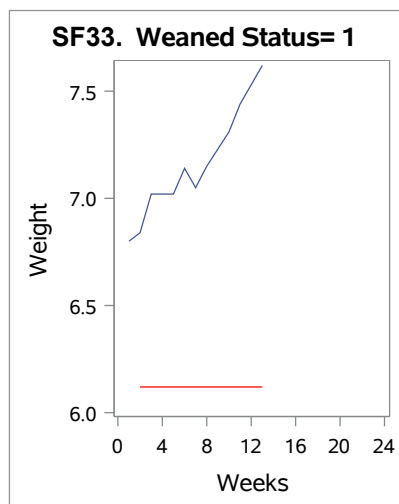

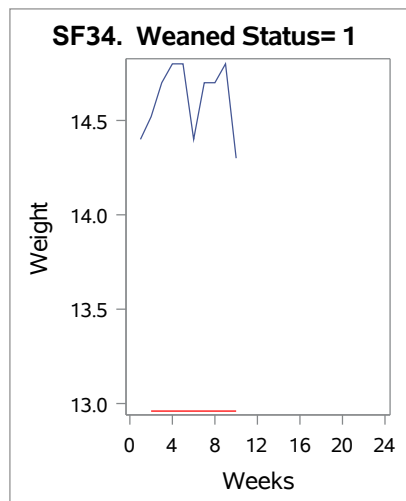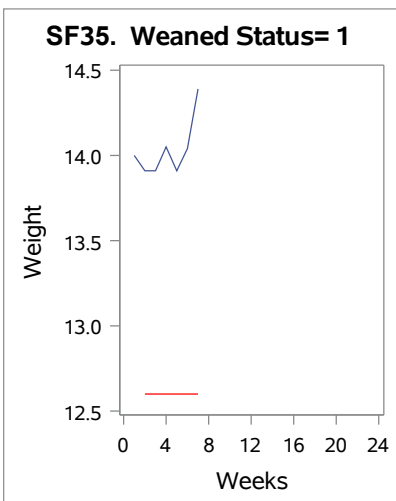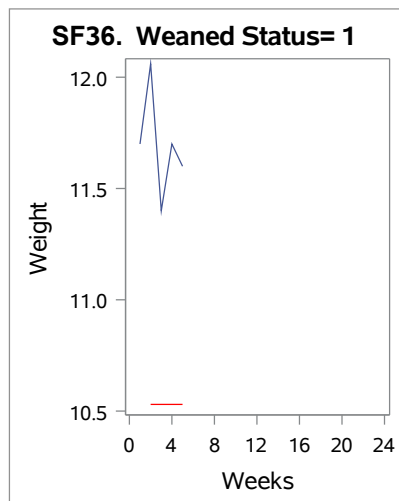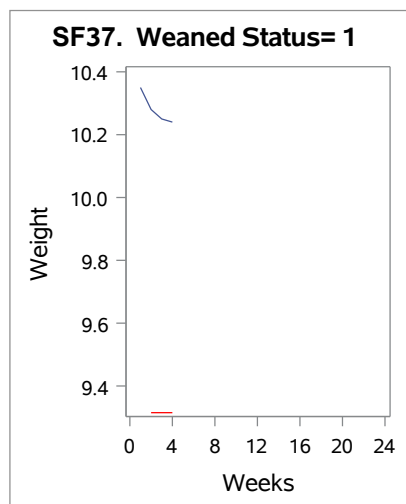

Supplement: Supplementary file 1 [file nutrients-18-00987-s001.zip › nutrients-4151471-Figure S1.pdf]
